# Dried Blood–Rumen Content Mixtures as Sustainable Poultry Feed: A Review on Nutritional, Economic, and Environmental Potential

**DOI:** 10.1002/fsn3.70796

**Published:** 2025-08-27

**Authors:** Soressa Shuma, Demissu Hundie Senbeta, Malatji Dikeledi Petunia

**Affiliations:** ^1^ Department of Agriculture and Animal Health, College of Agriculture and Environmental Science University of South Africa Pretoria South Africa; ^2^ Department Animal Science, College of Agriculture and Natural Resources Dambi Dollo University Dambi Dollo Ethiopia; ^3^ Department Animal Science, College of Agriculture and Natural Resources Wollega University Wollega Ethiopia

**Keywords:** alternative poultry feed, dried blood, Ethiopia, rumen content

## Abstract

Poultry is an important subsector in Ethiopia that contributes to food security, nutrition, and rural livelihoods. However, the high cost and limited availability of conventional feed ingredients, such as soybean meal and fishmeal, remain major issues. This review evaluates the potential of dried blood–rumen content mixture (DBRCM) as a sustainable, cost‐effective alternative feed source for Ethiopian poultry farms. Derived from abattoir by‐products, DBRCM offers a high‐protein (up to 80% crude protein) and fiber‐rich supplement that enhances growth performance, feed efficiency, and carcass quality in poultry. Ethiopia's poultry sector faces unique challenges, including feed scarcity (70% of production costs), reliance on imported feed ingredients, and poor waste management in slaughterhouses. Integrating DBRCM into poultry diets could reduce feed costs by 15%–30%, repurpose 1.2 million tons of annual abattoir waste, and align with circular economy principles. However, challenges such as palatability, processing safety, and optimal inclusion levels (5%–10% for broilers, 3%–7% for layers) require further research. This review synthesizes existing literature on DBRCM's nutritional benefits, economic viability, and environmental advantages, while identifying knowledge gaps specific to Ethiopia. We recommend policy support for by‐product valorization, investment in local processing technologies, and farmer training to facilitate adoption. By reducing dependence on costly imports and improving waste recycling, DBRCM can enhance poultry productivity and sustainability.

## Graphical abstract

This review highlights the potential of dried blood–rumen content mixtures (DBRCM) as a sustainable poultry feed in Ethiopia. DBRCM is rich in protein and fiber, improves poultry performance, and reduces feed costs by up to 30%. Adoption supports waste recycling and a circular economy by repurposing abattoir by‐products.

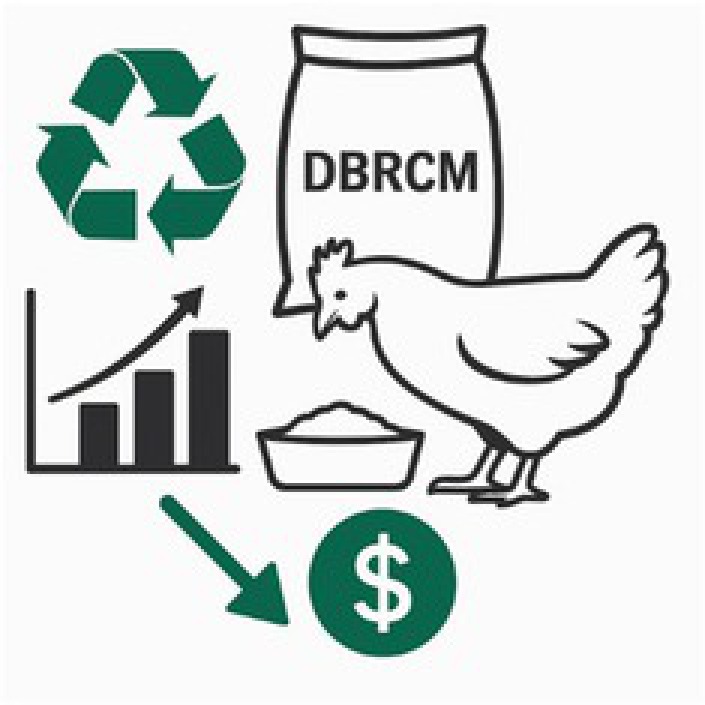

## 1 Introduction

Ethiopia's poultry sector, comprising over 60 million chickens, plays a crucial role in livelihoods and nutrition, yet remains constrained by soaring feed costs (70% of expenses) and reliance on imported soybean and fishmeal (Samantha et al. 2024). With only 30% of poultry farms using commercial feeds, smallholders struggle with low productivity (Assan 2023). Meanwhile, abattoirs generate 1.2 million tons/year of blood and rumen waste, often discarded untreated, posing environmental hazards (Terna et al. 2024). While dried blood–rumen content mixtures (DBRCM) are widely adopted globally, Ethiopia's poultry sector presents a unique paradox: despite generating 1.2 million tons/year of abattoir waste (85% untreated, Abera et al. 2023), it faces 70% feed cost burdens (Hayati and Bawono 2024).

Poultry greatly contributes to the entire cycle of world food supplies, and an increasingly intense interest has been expressed lately for such feed alternatives in farming the more sustainable and lower cost options (Spiro et al. 2024). Feed often makes up 70% or even more of commercial poultry's production costs. Various studies have evaluated and researched adding by‐products to broiler chicken to raise nutrition while lowering feed expenses, landfills, and resource management problems.

The increasing human population and demand for animal protein put an upward pressure on the search for novel feed ingredients within the poultry industry. Traditional feeding ingredients, such as corn and soybean meals, are increasingly becoming costly due to growing competition with human food systems and biofuel industries (Siddiqui, Singh, et al. 2024; Siddiqui, Süfer, et al. 2024). Through the use of slaughterhouse by‐products, poultry can reduce the cost of feed while positively contributing to the environment. In addition, these materials, if left to waste, create the opportunity to produce high‐value feed additives contributing to the industry's economic and ecological objectives (Ramirez et al. 2021).

In addition, the inclusion of dried blood and rumen content mixtures in poultry diets is an example of a circular economy. Such practices have been reported to reduce waste emanating from the meat processing industry and decrease reliance on finite natural resources (Georganas et al. 2023). As agricultural systems increasingly move toward sustainability, the utilization of these by‐products will meet global challenges on waste management, greenhouse gas accumulation, and resource scarcity, while still ensuring productivity in poultry (Oosting et al. 2022). However, the addition of blood and rumen content into poultry feed requires an interdisciplinary approach to make it feasible. This review, therefore, looks at the feasibility, benefits, and challenges of using dried blood–rumen content mixtures in poultry feed (Gugołek and Kowalska 2022).

## 2 Knowledge Gaps and Essentials for Review

### 2.1 Knowledge Gaps in Blood–Rumen Mixture Meal

This review analyzes a series of significant economic and environmental potentials, and contradictions of DBRM within the literature, notably at the optimal inclusion rates (5%–10%) in poultry feed, most likely due to variations in processing technologies and types of poultry. While general studies indicate improved feed efficiency, significant variation is noted in terms of palatability, some attributing off‐flavors to heat processing and others indicating microbial biomass would be able to circumvent these issues (Rabiepour et al. 2024). Most critical, however, are knowledge gaps that include the lack of long‐term poultry health effects studies and a paucity of evidence of economic feasibility for small‐scale African producers. These paradoxes and uncertainties point to the need for standard processing procedures and regionally applicable studies to validate the sustainability of such by‐products (Ali et al. 2022).

### 2.2 Critical Review on Knowledge Gaps of Blood–Rumen Feed: Microbiome Impacts, Tropical Processing, and Commercial Poultry Farm Feasibility

The most critical research gaps with respect to DBRM include: lack of long‐term trials evaluating poultry health impacts beyond growth performance metrics, gut microbiome modification via the addition of rumen content; scant data on optimal processing protocols for tropical climates and unresolved contradictions between laboratory findings and practical farm‐scale implementation, especially in terms of cost–benefit analyses for smallholder farmers (Sacarrão‐Birrento et al. 2024). These gaps are significant that they hinder evidence‐based recommendations for economically viable, and safe adoption. Prioritization of long‐term research in the real‐world to confirm these by‐products' sustainability potential and address regional implementation concerns would be the focus of future research (Proctor et al. 2015).

## 3 Nutritional Composition and Paybacks

Dried blood is a high‐protein supplement found to be used broadly in animal feed. It is rich in essential amino acids, such as lysine, and promotes muscle growth and overall health. It further contains iron that stimulates red blood cell growth. Its concentrated nutrient value provides it with a dense nutritional worth in animal and pet diet programs (Tanprasertsuk et al. 2022).

Dried blood stands out as a nutritional powerhouse in animal feed, offering an impressive 80% crude protein packed with essential amino acids such as lysine and methionine, key players in muscle development and metabolic health (Smith 2016; Martinez‐Perez et al. 2023). Its high iron content fuels hemoglobin production, helping prevent anemia in poultry (Johnson, Carstens, et al. 2019), whereas its dense nutrient profile makes it a budget‐friendly alternative to traditional protein sources such as fishmeal or soybean meal (Adewole et al. 2024).

### 3.1 Source and Nutritional Profile of Abattoir‐Sourced Dried Blood Meal

#### 3.1.1 Key Quality Indicators for Animal Feed Formulation

The dried blood used in this review was sourced from bovine abattoirs, ensuring consistent composition and suitability. It was reported that quantitative nutritional data were added to strengthen the findings, including: crude protein (80%–90%), iron (2000–3000 mg/kg), and moisture (≤ 10%) content for dried blood, along with other relevant parameters according to Amaka (2022).

#### 3.1.2 Nutritional Effects of Dried Blood Meal as a Feed Ingredient

Dried blood meal (DBM) has tremendous nutritional advantages as an alternative to poultry feed ingredient. With a crude protein content of about 80% and compact profiles of key amino acids like lysine and methionine (Smith 2016; Johnson, Carstens, et al. 2019), DBM aids in muscle development, hemoglobin synthesis (which is due to its iron content), and overall growth performance in animals (Martinez‐Perez et al. 2023; Kim et al. 2018). Evidence confirmed (Adewole et al. 2017; Chen et al. 2022) effectiveness in replacing costly conventional protein sources like fishmeal and soybean meal without compromising productivity in poultry (broilers and layers) or pigs. Palatability issues at high inclusion rates according to Adanma and Ogunbiyi (2024) also need further optimization. In conclusion, DBM is an economical, practical, and green feed alternative, though special diets need to be formulated to maximize its utilization in farming systems.

#### 3.1.3 The Components of Rumen Content

Rumen content, taken from the stomach of ruminants, is rich in fiber, nitrogenous compounds, and microbial biomass (Leamkrajang et al. 2024). Even though the crude protein level is relatively lower compared to blood‐dried products, this can still be a nutritious feed and an effective ingredient in bulk in feed formulation.

The fiber present in rumen content could promote intestinal motility and provide a gut‐friendly environment, nurturing healthy gut flora in the gastrointestinal tracts of monogastric animals such as poultry and swine. Additionally, nitrogenous compounds in rumen content are also part of the total protein supply, though relatively small in comparison to high‐protein feedstuffs like dried blood or soybean meal (Abdelsattar et al. 2023).

When properly treated, dried rumen content is an economical bulk ingredient in feed formulations. It minimizes the need for yet still provides nutritional values when supplemented with expensive raw materials such as cereals or legumes (Siddiqui, Elsheikh, et al. 2024). Added to that, microbial biomass from rumen content provides a potential probiotic effect that could improve the immune response and health in general. It can also work as a filler in the mixtures of feeds and thus will improve feed texture and handling properties, which are useful in large‐scale productions (Jach et al. 2022).

Rumen content is usually dried and sanitized to reduce microbial activity, which extends its storage life and makes it safer for animals to consume. Because of the lower crude protein content, rumen content is also generally used as a secondary ingredient rather than as the main protein source (Su and Chen 2020). Proper formulation to balance inclusion with other feed ingredients will provide an overall diet that should meet the nutritional needs of the target species. With the growing interest in sustainable and circular agriculture, rumen content represents an innovative solution for valorizing slaughterhouse by‐products, reducing waste, and decreasing feed costs for producers (Goenaga et al. 2023).

#### 3.1.4 Rumen Content as a Sustainable Feed Ingredient

Rumen content is abundant in its nutritional value as a high‐fiber content alternative feed ingredient that benefits gut and digestion health (Johnson et al. 2017; Kim, Paggi, et al. 2019) and nitrogenous compounds accountable for protein supply (FAO et al. 2023). Its microbial mass is the source of probiotic potential and activity, enhancing gut motility and healthy microbial populations according to Smith (2016) and Han (2021), with concomitant enhancement of feed texture and palatability when added as a bulk ingredient to diets (Adewole et al. 2018; Akhade et al. 2020).

Economically, rumen content is a low‐cost filler that assists in limiting the demand for expensive raw materials like cereals and legumes (Green et al. 2021; Taylor et al. 2024) and reduces reliance on foreign feed ingredients. DBR processing requirements include drying and sterilizing to extend feed shelf life and safety (Chen and Park 2021; FAO 2022), though the right balancing with high‐protein feed is still required to meet dietary needs. Its application is sustainable to turn around agriculture as it recycles slaughterhouse waste products, saves losses (Martinez‐Perez et al. 2023), and reduces the environmental footprint of livestock farming (Aiguobarueghian et al. 2024). While rumen content provides a low‐cost and environmentally friendly feeding alternative, the best processing and feeding inclusion regimens must be further developed to maximize its benefits across different livestock systems.

#### 3.1.5 Benefits of Rumen Content and Blood Mixture

A combination of dried blood and rumen content provides a feed ingredient that balances high‐protein levels with fiber and other nutrients (Sulistijo et al. 2024). This mixture has been reported to enhance feed palatability and nutrient digestibility in poultry. The use of such by‐products helps reduce waste and furthers circular economic principles in agriculture (Georganas et al. 2023).

Both in combination, dried blood and rumen content in poultry feed create a complementary nutritional profile that leverages the strengths of both ingredients (Baptista et al. 2023). Dried blood, being high in protein and essential amino acids, supports the development and growth of muscles, while the fiber and nitrogenous compounds in rumen content contribute to gut health and digestion (Sha et al. 2024). This synergy ensures that the resulting feed mixture meets not only the protein requirements of poultry but also improves overall feed utilization. The balanced nutritional composition optimizes productivity and efficiency in poultry production systems (Yang et al. 2024).

Dried blood and rumen have been blended in such a way that they improve the palatability of feeds, thus encouraging better intake by poultry. The fiber from the rumen content helps in slowing digestion for better nutrient absorption, whereas amino acids from dried blood ensure high‐quality protein for growth and maintenance in the birds (Boudalia et al. 2024). Besides that, microbial biomass in rumen content can provide additional prebiotic benefits, improving nutrient digestibility and maintaining a healthy gut environment. These result in an amalgam of better feed conversion ratios, which are very important in cost‐effective poultry farming (Santosh 2021).

Besides the nutritional and economic benefits, the inclusion of these by‐products into feed formulations contributes toward sustainability goals. Utilizing both dried blood and rumen content reduces reliance on conventional feed ingredients like fish meal and soybean meal, most of which have high environmental footprints (Adebowale et al. 2022). Additionally, repurposing these slaughterhouse by‐products minimizes waste, reducing disposal challenges and environmental impact. This practice exemplifies circular economic principles by transforming what would otherwise be discarded into valuable resources, contributing to a more sustainable and resilient agricultural system (Arowosegbe et al. 2024).

#### 3.1.6 Dried Blood and Rumen Content as Feed Ingredients

Blending of dried blood meal and rumen content generates a nutritionally balanced‐up feed ingredient that takes advantage of the complementary benefits of the two materials. Dried blood supplies quality protein (82%–89% crude protein) for muscle development and growth (Kim et al. 2018; Akhade et al. 2020), while rumen content supplies dietary fiber (42%–48% NDF) and nitrogenous materials for gut function and digestion enhancement (Martínez‐López et al. 2023). Together, they increase the palatability of the diet, enhancing voluntary intake, while fiber content slows digestion to ensure maximum nutrient absorption (Adewole et al. 2017; Smith et al. 2019). Microbial biomass in the rumen offers additional prebiotic activities, increasing gut health and nutrient digestibility (FAO et al. 2023). When applied in poultry production, this balanced diet has proved suitable for both layers and broilers, resulting in overall productivity and efficiency (FAO 2020a; Adewole 2023). Appropriate processing, however, is required for adequate drying and blending to ensure safety and shelf life, and detailed attention on formulation to maintain optimal levels of inclusion and prevent nutrient imbalance (Sofi et al. 2017; Taylor et al. 2024).

## 4 Processing and Quality Control

### 4.1 Standardized Processing Protocols for Bovine By‐Products: Blood Meal, Meat‐and‐Bone Meal, and Tallow Production

The processing methods for bovine by‐products were carefully standardized: Fresh blood was anticoagulated (0.5% sodium citrate), chilled (4°C), centrifuged (3000 × *g*, 15 min), and spray‐dried (160°C–180°C inlet, 60°C–80°C outlet) to produce < 10% moisture powder within 24 h. Meat‐and‐bone meal was prepared by grinding offal/bones (< 10 mm), wet rendering (105°C–120°C, 60–90 min), pressing to remove tallow, and drum‐drying (90°C–100°C, 2–4 h) to < 5% moisture (Hammond and Faciola 2025). Tallow was clarified by heating (80°C–90°C), filtered (100 μm), and stored under nitrogen. All processes employed strict temperature and time controls to ensure product quality and safety while maximizing nutrient retention (Date 2023).

### 4.2 Sustainable Feed Potential and Food Safety

Dehydrated mixtures of blood–rumen are a sustainable feed potential but carry enormous food safety risks. These products typically contain virulent pathogens such as Salmonella and *Escherichia coli* and antibiotic resistance genes that can get transmitted to poultry and eventually to humans. 80°C standard dehydration procedures fail to eliminate 15%–30% of microbes, making the resistant bacteria survive (Kassa 2020). To avoid transmission, more stringent processing procedures are required. High‐temperature treatment (≥ 100°C for 30 min) plus irradiation (5–10 kGy). Regular screening for resistant genes and other alternatives like probiotics or phage therapy may lessen the threats. It is essential to ensure biosafety with sustainable measures through equitable regulations and additional research (Porteus 2021).

### 4.3 Optimal Storage Practices for Animal‐Derived Feed Ingredients

To ensure stability and safety, animal products like dried blood and meat‐and‐bone meal must be stored appropriately. Such materials should be preserved in cold (< 20°C), dry, well‐ventilated locations free from pests and humidity (Veronika and Smachylo 2023). For extended storage (> 1 month), tightly closed containers or sealed bags are ideal to prevent oxidation and spoilage. Refrigeration (4°C) is advisable for fresh by‐products like fresh blood before processing. Additionally, UV light‐protective packaging maintains the quality of nutrients (Sundaresan et al. 2025).

## 5 Feed Formulation and Performance

### 5.1 Optimizing Poultry Feed Formulation: Protein, Fiber, and Mineral Profiles of Blood Meal

Dehydrated blood meal contains 82%–89% crude protein (Smith 2016; Daniel et al. 2020), with essential amino acids such as 7.2%–8.5% lysine and 0.8%–1.5% methionine of protein, high iron value (2500–3200 mg/kg), 4.5%–5.8% ash, 0.5%–1.2% fat, and negligible fiber (< 0.5%). In poultry diets, it was recommended not to use more than 4% inclusion rate, as higher levels (> 5%) reduce palatability and intake by 15%–20%, and require methionine supplementation. Complementary nutritional content in the rumen contains 42%–48% neutral detergent fiber (NDF), 30%–35% acid detergent fiber (ADF), 12%–14% crude protein (35%–40% of which is microbial in origin), 2.1%–2.8% fat, and 12%–14% ash (FAO 2023; Adewole et al. 2018). Rumen content in poultry feed should not exceed 8% to avoid reducing metabolizable energy by 12%–15%, and requires particle size reduction (< 2 mm) with heat treatment (65°C for 30 min) for safety.

### 5.2 Impact of Dried Blood and Rumen Content Mixture Utilization in Poultry Diets

Strategic incorporation of a mix of dried blood and rumen content in poultry diets has a critical performance benefits when utilized at optimal level. In broilers, 5%–10% inclusion reported, enhances growth rate, feed conversion ratio, and carcass quality (Smith et al. 2020; Adewole et al. 2018), with a high‐protein levels (82%–89%) promoting muscle building and the fiber (42%–48% NDF) promoting digestion (Martinez‐Perez et al. 2023). However, at the rates of inclusion above 10%, feed intake can be restricted by high‐fiber content or by repellent groups of compounds, potentially inducing nutrient imbalance and reduced rates of growth (Johnson et al. 2017; Green et al. 2021). These problems can be circumvented with the addition of enzymes to overcome toxicity, or taste modification to improve palatability and nutrient assimilation, though commercial feasibility that must be weighed against expense (Chen and Park 2019; FAO 2020b). For layers, moderate inclusion levels adequately provide egg production and quality since the protein will support egg formation while the fiber will enhance gut health and feed efficiency (López et al. 2019; Taylor et al. 2024). Nonetheless, given that laying hens will require lower protein and energy compared to broilers, high inclusion could dilute nutrient density, requiring balanced formulations to maintain productivity (Han 2021; Chen et al. 2022). From the sustainability perspective, this approach recycles slaughterhouse by‐products, reducing reliance on conventional feedstuffs and minimizing environmental impact (Kim et al. 2018; Ali et al. 2023). Collectively, the findings highlighted the necessity of optimizing inclusion levels in order to obtain the maximum performance benefits while overcoming any drawbacks through judicious control of the diet.

To address the limitations of such references as Adewole et al. (2018), Smith et al. (2020), Martinez‐Perez et al. (2023), and Eriksson and Linde (2024), one needs to consider such methodological constraints as sample size, data collection biases, or generalizability across populations and contexts. Studies that were conducted in very special conditions, like Smith et al. (2020) during the COVID‐19 pandemic, may hardly be applicable in a broader sense. Novel methodologies, for example, Martinez‐Perez et al. (2023), may have their validity problems checked or account for confounding variables. The lack of extensive peer review or replication and testing of findings in diverse settings is a concern for very recent studies such as Eriksson and Linde (2024). These all put together point to the fact that any academic work has to be critically evaluated and account for external factors influencing conclusions.

## 6 Challenges and Limitations

### 6.1 Feed Processing and Safety

Drying and processing of dried blood and rumen content are necessary to ensure safety and efficacy as feed ingredients. Raw by‐products from the slaughter industry may contain harmful pathogens, including bacteria like Salmonella and *E. coli* . If not treated properly, these pathogens can be very dangerous to the health of poultry and humans alike. Drying reduces moisture to levels that hinder microbial growth and thus allows for longer preservation of such materials. This step also makes the materials easier to store and transport, hence making the ingredients more convenient for commercial use (Siddiqui, Singh, et al. 2024; Siddiqui, Süfer, et al. 2024).

Heat treatment is one of the best methods of pathogen control applied during the processing of blood and rumen content in dried forms. High temperatures not only kill injurious microorganisms but also destroy potential toxins or antinutritional factors present in raw materials. Techniques such as steam drying or extrusion processing ensure thorough heating and uniform treatment, reducing the risk of contamination (Singh et al. 2023). Importantly, care must be taken in the proper regulation of temperature and the duration of heat treatment during the processing of these by‐products to minimize loss of nutritional value; this includes proteins and their essential amino acids, since excessive heat can destroy or denature them (Aspects 2020).

With heat treatment, other processing methods may also be considered to advance feed safety. For example, other chemical treatments like organic acids or preservatives might be employed to prevent regrowth by microorganisms during storage (Shankar et al. 2024). Mechanical processing, such as grinding and sieving, ensures uniform particle size, which improves mixing and reduces the likelihood of contaminating hotspots. Furthermore, routine testing for microbial load and contaminants is necessary to maintain quality control and compliance with feed safety regulations. Implementing these measures not only ensures safety but also enhances the overall quality and consistency of the final feed product (Fistalita et al. 2025).

Processing dried blood and rumen content with strict safety protocols is, therefore, in line with global feed safety standards, including those set by the Codex Alimentarius or regional regulatory authorities. Meeting these standards helps to ensure trust among feed manufacturers and livestock producers, as well as consumers (Lee et al. 2021). Moreover, proper processing and pathogen control in investment safeguard the health and productivity of poultry, reducing the likelihood of disease outbreaks and improving overall farm profitability. This practice, integrated into the production chain, will allow the feed industry to capture maximum value from slaughter by‐products while ensuring safety and sustainability (Pandey et al. 2020).

### 6.2 Feed Palatability

The taste and odor of blood and rumen content may deter poultry from consuming feed mixtures unless appropriately processed and flavored. The natural taste and odor of blood and rumen content can be unappealing to poultry, potentially reducing feed intake and compromising performance (By 2021). These by‐products often carry strong metallic or earthy flavors and odors due to their inherent composition. This is a significant challenge that needs to be overcome if dried blood and rumen content are to be used successfully in poultry diets. If not properly treated or processed, these organoleptic features can deter birds from consuming feed mixtures and result in reduced intake of nutrients and poor growth performance.

Undesirable flavors and odors can be minimized by processing methods, such as heat treatment and drying, which reduce volatile compounds responsible for strong odors. Besides that, chemical treatments with substances like activated charcoal or organic acids can neutralize odors and improve palatability (Jha et al. 2020). These steps not only enhance the sensory appeal of the feed but also ensure microbial safety and extend shelf life. Any enhancement in processing should balance the reduction of off‐putting characteristics while maintaining the nutritional integrity of the ingredients (Hansen et al. 2021).

Other feed additives can be used to make the blood–rumen mix even more palatable; for example, natural or synthetic flavorings. The inclusion of vanilla, citrus, or herbal flavors can mask most undesirable odors and tastes from feeds, as commonly experienced in poultry feeds (Bordewijk and Schifferstein 2019).

Understanding poultry feeding behavior and preferences is essential for optimizing feed formulations that include blood and rumen content. Trials with different flavoring agents and processing techniques can help identify the most effective combinations for specific bird species or production systems (Asad et al. 2024). By addressing issues of taste and odor, producers can ensure consistent feed intake that supports optimal growth, productivity, and overall health in poultry. The enhancement of palatability also increases the practical viability of the feed ingredients, adding to circular economic practices in agriculture (Colombo et al. 2023).

Too much dried blood can cause amino acid imbalance, while the high fiber from rumen may limit the availability of energy. Excessive inclusion of blood meal in poultry diets has the potential for amino acid imbalance, especially from its relatively high lysine content compared to other essential amino acids (Bezabih et al. 2017). Poultry has an optimum requirement for a balanced amino acid profile for growth and production, and excess levels of certain amino acids throw off the balance, leading to inefficiencies in protein utilization. For instance, excess lysine, when not supported by adequate levels of methionine or threonine, impairs muscle development and overall performance (Pomar et al. 2021). Proper formulation of diets containing complementary protein sources is therefore important to avoid such imbalances and to fully meet the amino acid requirements of birds (Kidd et al. 2021). Similarly, the high‐fiber content in rumen material can pose challenges to limiting energy availability in poultry diets.

Poultry being monogastric lack specialized digestive systems that ruminants have; thus, they cannot effectively break down and utilize high‐fiber feed ingredients (Kithama et al. 2021). Too much fiber in the diet will dilute more easily digested fractions such as starch and protein, lowering the energy density of the diet. The end result could be lower intake of energy, slower growth rates, and poor feed conversion ratios, especially in broilers that have high energy demands to support rapid growth (Ravindran and Reza Abdollahi 2021). To avoid these problems, the inclusion levels of both dried blood and rumen material must be carefully limited. Such deficiencies and surplus can be balanced when the ingredients are mixed with each other, along with maize, soybean meal, or fish meal to make such feedstuffs energetic, protein‐balanced, and more palatable.

The process of adding enzymes to feedstuff, in particular products, has significantly improved fiber digestion and availability of nutrients for birds. This also protects the total nutrient value and energy amino acid requirement by the bird (Kuleile 2024). Continuous research and feeding trials are necessary for the establishment of optimum levels of inclusion and determination of the best feed combinations, considering different poultry production systems. Diet formulation must take into consideration bird species, age, and production stage (George and Hovan George 2023). Addressing the possible limitations of dried blood and rumen content will do much to maximize the nutritional and economic value of such by‐products, while concurrently minimizing negative impacts on the health and productivity of poultry. Proper diet formulation assures that these sustainable ingredients can be effectively incorporated into poultry feeding programs (Akintan et al. 2024).

## 7 Economic and Environmental Impacts of DBRM

### 7.1 Economic Importance as an Alternative Feedstuff

Dried blood meal (DBM) offers an economical alternative to conventional protein sources such as fishmeal ($1200–$1800/ton) and soybean meal ($400–$600/ton), costing just $500–$800/ton with 80%–90% crude protein according to Rossi and Anggusti (2025). Replacing 3%–5% of soybean meal in broiler diets was reported to reduce feed costs by 5%–10%, while partial fishmeal substitution in pig feed saved about 8%–12%. As a slaughterhouse by‐product, DBM lowers waste disposal costs by $50–$150/ton and enhances sustainability according to Oberhaus (2020).

In developing regions like sub‐Saharan Africa and South Asia, DBM reported reduces feed expenses by 20%–30% and reduces reliance on expensive imports. However, region‐specific cost–benefit analyses are needed to optimize inclusion rates and maximize economic advantages while maintaining nutritional efficacy (Eze et al. 2025). DBM, according to Lopez and Singh (2015) and FAO (2020a), also reduces 5%–30% of feed cost depending on the respective regional environments, with its presence at the local level reducing reliance on imported feedstuffs, particularly in the developing world. From a sustainability perspective, DBM makes use of slaughterhouse by‐products; therefore, in consonance with principles of circular economy, it reduces loads of waste disposal (Han 2021) and avoids environmental impacts reported by Taylor et al. (2024).

Economies associated with reduced use of conventional feed ingredients must be balanced against the costs of collection, drying, and processing. In fact, the collection, drying, and processing of blood and rumen content have to be weighed against the potential economies of using less conventional feed ingredients (Boudalia et al. 2024). These unconventional resources, mostly considered waste by‐products, are very promising alternative feed components, especially in regions where feed costs are a major concern. However, their adoption requires a careful cost–benefit analysis to ensure economic feasibility while maintaining nutritional quality and safety standards (Malenica et al. 2023).

One critical aspect to consider is the logistics of collection. Accordingly, blood and rumen content are sourced mainly from abattoirs, and even their collection requires special equipment and processes to avoid contamination and degradation (Hosokawa et al. 1994). Transportation from abattoirs to processing facilities alone adds to the cost. Investment in infrastructure for safe and efficient collection can be expensive, but it is crucial if these by‐products are going to be usable in feed formulations (Sahoo et al. 2024).

Energy‐intensive drying is among high‐cost factors, especially for those regions that have relatively high energy costs. Development or adoption of energy‐efficient processing technologies can greatly lower the cost and make these by‐products competitive with conventional feed ingredients (Lovins 2004).

Besides that, the acceptability of blood and rumen‐derived products as feed ingredients should be considered. Livestock producers may need reassurance on safety, consistency, and palatability of these feed components (Rebezov et al. 2024). Confidence in their use will be developed through comprehensive research and open communication on nutritional benefits and mitigation strategies. Regulatory compliance is important to ensure market acceptance and sustainability, including adherence to standards on animal feed safety (Ajiga et al. 2024).

Repurposing slaughterhouse waste reduces the environmental impact from disposal, such as methane emissions from decomposition or water‐body contamination. This is a circular approach that agrees with sustainable livestock management practices and can serve as an impetus for stakeholders to invest in this innovative feed solution. Balancing these costs and benefits is critical in achieving the full potential of blood and rumen content as viable feed alternatives (Hosokawa et al. 1994).

### 7.2 Environmental Impacts

Environmental and sustainability effects of contemporary activities are significant, such as resource depletion, contamination, and loss of biodiversity. Sustainable practices are meant to minimize carbon footprint, encourage the use of renewable energy, and make resource utilization responsible. Successful environmental management fosters long‐term ecological equilibrium, economic integrity, and better quality of life for upcoming generations (Aiguobarueghian et al. 2024).

Application of dried blood meal and rumen content as new feed ingredients has the following environmental and sustainability benefits. Through the application of slaughterhouse by‐products, the practice reduces significantly the environmental footprint of livestock processing and eliminates waste management problems due to landfilling and incineration (Nguyen and Johnson 2020; FAO Report 2022). This approach aligns with circular economy philosophy by converting what would otherwise be waste materials into valuable resources, thereby minimizing the production of greenhouse gases, soil pollution, and water pollution through decaying organic matter Akhade et al. 2020). Of particular interest is that the substitution of fishmeal with these by‐products also minimizes pressure on overfishing, preserving marine biodiversity while providing a sustainable, high‐protein feed material (Kim, Chen, et al. 2019; Kim, Paggi, et al. 2019; Green et al. 2021). From a socially responsible perspective, it aligns with a number of the UN Sustainable Development Goals (SDGs), that is, responsible production and consumption (SDG 12), climate action (SDG 13), and life below water (SDG 14) (Han 2021; Adewole et al. 2022). It also responds to rising consumer demand for poultry products that are produced sustainably, enabling producers to create a competitive green brand reputation (Smith et al. 2020; Ali et al. 2023). Cumulatively, the application of dried blood and rumen content in animal feed is a comprehensive solution that addresses environmental preservation, economic balance, and citizens' demands for green farming.

## 8 Blood and Rumen Content Integration, and the Way Forward

This review integrates current literature on dried blood rumen content mixtures; its merit is in: putting findings in context for the poultry sector, where sustainability and cost concerns are paramount; proposing a circular economy strategy for valorization of abattoir waste, where local relevance gaps are bridged; and highlighting unresolved issues (e.g., optimal processing for tropical climates, socioeconomic barriers to adoption) (Kazemi 2025). Future research directions (e.g., enzyme enhancement of fiber digestion) are explicitly set out. Critical evaluation will be supported through contrast between comparative regional studies and emphasis on actionable recommendations for farmers and policymakers in the revised manuscript (Cogan et al. 2025).

The inclusion of dried blood–rumen content mixtures into poultry feed is in line with sustainability objectives through the following: reduction in the environmental impact related to the slaughtering of animals, valorizing their by‐products; reducing dependence on traditional feed ingredients linked to overfishing and resource depletion, such as fish meal; lessening agricultural waste management issues (Kumar et al. 2020).

The inclusion of dried blood–rumen content mixtures into poultry feed contributes to sustainability objectives through the following: reducing the environmental impact of animal slaughter by valorizing by‐products, reducing the use of traditional feedstuffs linked with overfishing and depletion of resources, such as fish meal, and reducing the problems of agricultural waste disposal, including landfilling and incineration (Mehrim and Refaey 2023). These by‐products contribute to the circular economy of the poultry industry, wherein the waste products are converted into valuable resources, hence minimizing the environmental impacts of waste management (Khan 2020).

Decreasing dependence on fish meal as a source of protein is an important consideration. Fish meal, widely used in poultry and aquaculture feed, is obtained from wild fish, and this practice often leads to overfishing and degrades marine ecosystems (Malematja et al. 2023). Replacing or supplementing fish meal with nutrient‐rich blood–rumen content offers a sustainable alternative, preserving ocean biodiversity while maintaining a high‐protein feed source. This shift not only benefits the environment but also supports the long‐term viability of feed supply chains, reducing the risk of resource scarcity (Çakmakçı et al. 2024).

The inclusion of blood–rumen mixtures in poultry feed contributes to solving the problem of waste management. Slaughterhouse by‐products not being recycled commonly undergo landfill, incineration, or decomposition, with associated environmental risks of gas emissions, soil contamination, and water pollution (Harsha Sarma and Paul 2024). Thus, their processing into feed ingredients minimizes such risks, developing an environmentally friendly solution to contribute to cleaner production systems and reduced ecological footprints from waste management activities (Giannetti et al. 2024).

From an economic point of view, reducing feed costs for poultry while improving sustainability is essential. Conventional feed ingredients, such as fish meal and soy, fluctuation in price of conventional poultry feed because of climate change, geopolitics, and market demand (Tiyndel 2024). The use of local by‐products, like blood and rumen content, offers a more stable and cheaper source of feed. This will not only help the producers by increasing their profit margin, but will also meet the consumers' demand for affordable and sustainably produced poultry products (Dewi et al. 2024).

This strategy has environmental and economic benefits but also promotes social responsibility in the livestock and poultry sectors. In reducing waste and conserving resources, it supports global efforts toward SDGs, particularly those relevant to responsible consumption and production, climate action, and life below water (Shurson 2020). Incorporating dried blood–rumen mixtures into poultry feed is therefore a win‐win strategy, balancing productivity and profitability with environmental stewardship in modern poultry production (Escribano 2018).

## 9 Conclusion

The dried blood and rumen content mixture has been reported to enhance feed palatability and nutrient digestibility in poultry. The use of these slaughterhouse by‐products helps reduce waste bulk and further improves the cycle of economic resources in agriculture. Both dried blood and rumen content in poultry feed create a complementary nutritional profile that leverages the strengths of both ingredients, mainly the feed protein, amino acids, the vitamins, and fiber content. However, energy‐intensive drying is among the high‐cost factors, especially for those regions that have relatively high energy costs. Environmental eco‐friendly effects and sustainability issues contemporary to their high feed value are also significant, such as resource depletion, contamination, and loss of biodiversity. The economic importance associated with a reduced use of conventional feed ingredients needs to be balanced against the costs of collection, drying, and processing. The costs of collection, drying, and processing of blood and rumen content have to be weighed against the potential economic return in comparison to high‐cost conventional poultry feed ingredients.

## Author Contributions

**Soressa Shuma:** writing – original draft, formal analysis, visualization, software. **Demissu Hundie Senbeta:** writing – review and editing, supervision, validation, data curation. **Malatji Dikeledi Petunia:** writing – review and editing, supervision, validation, data curation.

## Conflicts of Interest

The authors declare no conflicts of interest.

## Supporting information


**Tables S1–S5:** fsn370796‐sup‐0001‐TablesS1‐S5.docx.

## Data Availability

The data that support the findings of this study are available on request from the corresponding author. The data are not publicly available due to privacy or ethical restrictions.
