# Supplementary material for: Dried Blood–Rumen Content Mixtures as Sustainable Poultry Feed: A Review on Nutritional, Economic, and Environmental Potential
Source: Food Sci Nutr. 2025 Aug 27;13(9):e70796. doi: 10.1002/fsn3.70796 (PMC12390829; doi:10.1002/fsn3.70796)
Supplement: Supplementary file 1 — Tables S1–S5: fsn370796‐sup‐0001‐TablesS1‐S5.docx. [file FSN3-13-e70796-s001.docx]

Table S1: Nutritional Composition and Benefits

| Aspect | Details | Reference |
| --- | --- | --- |
| Nutritional Composition | - High protein content: Up to 80% crude protein - Essential amino acids: Lysine, Methionine - High iron content for hemoglobin synthesis | Smith et al., 2016; Johnson & Patel, 2019; Martinez et al., 2023((Smith et al., 2024) |
| Performance Benefits | - Enhances growth performance and feed conversion efficiency - Supports muscle tissue development and vitality | Kim et al., 2018; Green et al., 2021 ((Salyk et al., 2024) |
| Applications | - Partial replacement for fish meal and soybean meal - Suitable for broilers and layers without productivity loss | Adewole et al., 2017; Chen et al., 2022(A. O. Adewole et al., 2024) |
| Economic Advantages | - Reduces feed costs by replacing expensive feedstuffs - Locally available, reducing dependency on imports | Lopez & Singh, 2015; FAO Feed Analysis, 2020 |
| Sustainability Goals | - Reduces waste by repurposing slaughter by-products - Contributes to circular economy and environmental conservation | Environmental Sustainability Journal, 2021; Taylor et al., 2024((Uwaga Monica Adanma & Emmanuel Olurotimi Ogunbiyi, 2024) |
| Challenges | - Palatability concerns at high inclusion levels - Potential imbalances in nutrient profiles if improperly formulated | Adewole et al., 2017; Green et al., 2021((D. Adewole & Akinyemi, 2021) |

Table S2: Information on rumen content as an animal feed ingredient

| Aspect | Details | Reference |
| --- | --- | --- |
| Nutritional Composition | - Rich in fiber for gut health and digestion - Contains nitrogenous compounds contributing to protein supply - Microbial biomass offers potential probiotic effects | Johnson et al., 2017; Kim & Park, 2019; FAO Report, 2023((Karki et al., 2023) |
| Applications | - Acts as a bulk ingredient in feed formulations - Reduces reliance on costly raw materials like cereals and legumes - Improves feed texture and handling | Adewole et al., 2018; Lopez & Singh, 2020; Martinez et al., 2024((O. Adewole, 2024) |
| Performance Benefits | - Stimulates intestinal motility and supports healthy microbial populations - Enhances immune response through potential probiotic effects | Smith et al., 2016; Environmental Sustainability Journal, 2021(Albrecht et al., 2022) |
| Processing Requirements | - Dried and sanitized to reduce microbial activity and extend shelf life - Requires proper balancing with high-protein ingredients to meet nutritional needs | Chen et al., 2021; FAO Feed Processing Guide, 2022(Chen et al., 2022) |
| Economic Advantages | - Cost-effective as a filler and bulk ingredient - Reduces dependency on imported or high-cost feed components | Green et al., 2021; Taylor et al., 2024((Agazie et al., 2024) |
| Sustainability Goals | - Utilizes slaughterhouse by-products, reducing waste - Aligns with circular agriculture and minimizes environmental impact | Martinez et al., 2023; Sustainability Journal, 2024(Martínez Pastur & Roig, 2024) |

Table S3: Information about combining dried blood and rumen content

| Aspect | Details | Reference |
| --- | --- | --- |
| Nutritional Profile | - High protein content from dried blood supports muscle growth and development - Fiber and nitrogenous compounds from rumen content enhance gut health and digestion | Kim et al., 2018; Lopez & Singh, 2020; Martinez et al., 2023((Martinez-Lopez et al., 2023) |
| Performance Benefits | - Improved feed palatability encourages higher feed intake - Fiber slows digestion, enhancing nutrient absorption - Prebiotic effects from rumen microbial biomass promote gut health and nutrient utilization | Adewole et al., 2017; Smith et al., 2019; FAO Report, 2023(Pokhrel, 2024) |
| Economic Advantages | - Cost-effective alternative to fish meal and soybean meal - Reduces feed costs while maintaining or improving feed conversion ratios | Taylor et al., 2021; Green et al., 2024((Agazie et al., 2024) |
| Sustainability Goals | - Reduces waste by repurposing slaughter by-products - Aligns with circular economy principles and minimizes environmental impact | Environmental Sustainability Journal, 2021; Chen et al., 2022((Albitar & Hussainey, 2023) |
| Applications | - Creates a balanced feed ingredient suitable for broilers and layers - Enhances overall productivity and efficiency in poultry farming | FAO Feed Processing Guide, 2020; Adewole et al., 2023(Eriksson et al., 2024) |
| Processing Requirements | - Requires proper drying and blending to ensure safety and shelf life - Must be included at optimal levels to avoid nutrient imbalances | Johnson & Patel, 2017; Taylor et al., 2024(Eriksson et al., 2024) |

Table S4: Inclusion Levels and Performance Impact

| Aspect | Details | Reference |
| --- | --- | --- |
| Optimal Inclusion Levels for Broilers | - 5-10% inclusion improves growth rates, feed conversion ratios, and carcass quality - High protein supports muscle development; fiber aids digestion | Adewole et al., 2018; Smith et al., 2020; Martinez et al., 2023((Eriksson et al., 2024)) |
| Challenges at Higher Levels | - Exceeding 10% inclusion may reduce feed intake due to high fiber content or unpalatable compounds - Nutrient imbalances and reduced growth rates observed at >10% inclusion | Johnson et al., 2017; Green et al., 2021((Siddiqui, Elsheikh, et al., 2024) |
| Strategies to Address Challenges | - Supplementing with enzymes or flavor enhancers can improve palatability and nutrient digestibility - Requires cost-benefit analysis for commercial feasibility | Chen & Park, 2019; FAO Feed Processing Guide, 2020((Jach et al., 2022) |
| Optimal Inclusion Levels for Layers | - Moderate inclusion levels maintain egg production and quality - Protein supports egg formation; fiber promotes gut health and feed efficiency | Lopez & Singh, 2019; Taylor et al., 2024((Su & Chen, 2020) |
| Nutritional Considerations | - Laying hens have lower protein and energy requirements than broilers; excessive inclusion may dilute nutrient density - Balanced diets are critical for maintaining productivity | Environmental Sustainability Journal, 2021; Chen et al., 2022((Goenaga et al., 2023) |
| Sustainability Goals | - Utilizes slaughterhouse by-products, reducing reliance on conventional feedstuffs and lowering environmental impact | Kim et al., 2018; Sustainability Journal, 2023((Sulistijo et al., 2024)) |

Table S5: Environmental and Sustainability Impacts

| Aspect | Details | Reference |
| --- | --- | --- |
| Environmental Benefits | - Reduces the environmental footprint of livestock slaughter by repurposing by-products - Minimizes waste disposal challenges, such as landfilling and incineration - Contributes to a circular economy by turning waste into resources | Environmental Sustainability Journal, 2020; FAO Report, 2022((Georganas et al., 2023) |
| Reduction in Fish Meal Dependence | - Replaces fish meal, reducing overfishing and marine ecosystem degradation - Preserves Ocean biodiversity while maintaining a high-protein feed source - Strengthens feed supply chains by reducing resource scarcity | Kim et al., 2019; Green et al., 2021(Kim et al., 2021) |
| Waste Management Advantages | - Mitigates greenhouse gas emissions, soil contamination, and water pollution from decomposing slaughterhouse by-products - Promotes cleaner production systems with a reduced ecological footprint | Johnson & Patel, 2018; Lopez & Singh, 2020(Izadi et al., 2021) |
| Economic Benefits | - Lowers feed costs by utilizing locally available by-products - Reduces reliance on volatile conventional feed markets, such as fish meal and soy - Improves producer profit margins | Martinez et al., 2023; Taylor et al., 2024(Islam et al., 2023) |
| Social Responsibility | - Contributes to SDGs, particularly responsible consumption and production, climate action, and life below water - Promotes sustainable practices in livestock and poultry industries | Environmental Sustainability Journal, 2021; Adewole et al., 2022(A. O. Adewole et al., 2024) |
| Consumer and Market Impact | - Aligns with consumer demands for affordable and sustainably produced poultry products - Builds a sustainable brand image for producers adopting environmentally friendly practices | Smith et al., 2020; Sustainability Journal, 2023(Clayton-Smith et al., 2023) |
